# Prognostic Value for Perioperative Serum Total Cholesterol Level on Postoperative Long-Term Prognosis of Pancreatic Cancer: A Retrospective Clinical Study

**DOI:** 10.3390/diagnostics13081402

**Published:** 2023-04-12

**Authors:** Han-Xuan Wang, Cheng Ding, Jin-Can Huang, You-Wei Ma, Shao-Cheng Lyu, Ren Lang

**Affiliations:** Department of Hepatobiliary and Pancreaticosplenic Surgery, Beijing Chaoyang Hospital, Capital Medical University, Beijing 100020, China

**Keywords:** pancreatic cancer, serum cholesterol level, predictive value, postoperative, long-term prognosis

## Abstract

Cholesterol correlates with occurrence and progression of pancreatic cancer and has predictive value for postoperative prognosis in various cancers. Our study intended to reveal the relationship between perioperative serum total cholesterol (TC) level and postoperative prognosis of pancreatic cancer. We retrospectively analyzed the data of pancreatic cancer patients who underwent surgical treatment at our hospital from January 2015 to December 2021. ROC curves between serum TC level at each time point and 1-year survival rate were drawn, from which study object and optimal cutoff value was determined. Patients were divided into low and high-TC groups, and perioperative data and prognosis were compared. Risk factors for poor postoperative prognosis were identified by univariate and multivariate analysis. Overall survival rates at postoperative 1, 2 and 3 years in the low and high-TC groups were 52.9%, 29.4%, and 15.6% and 80.4%, 47.2%, and 33.8% (*p* = 0.005), respectively. Multivariate analysis confirmed tumor differentiation degree (*RR* = 2.054, 95% CI: 1.396–3.025), pTNM stage (*RR* = 1.595, 95% CI: 1.020–2.494), lymph node metastasis (*RR* = 1.693, 95% CI: 1.127–2.544), and postoperative 4-week serum TC level (*RR* = 0.663, 95% CI: 0.466–0.944) as independent risk factors for prognosis of pancreatic cancer. We conclude that postoperative 4-week serum TC level has certain predictive value for long-term postoperative prognosis of pancreatic cancer.

## 1. Introduction

Pancreatic cancer is one of the most common gastrointestinal malignancies, whose incidence ranks 10th among all malignancies and has increased at an annual rate of 0.5–1% in recent years [1,2]. It has a high degree of malignancy. Pancreatic cancer is the fourth-leading cause of cancer-related death in the United States, and is expected to become the second-leading cause of cancer-related death in the United States by 2030 [3]. In 2023, the estimated number of deaths caused by pancreatic cancer will account for 8% of all tumor-induced deaths, ranking fourth among all tumors. The 5-year survival rate of pancreatic cancer is also unsatisfying—only 11% according to research [4]. In spite of the highly developed adjuvant therapy at present, radical surgery is still the only possible cure for pancreatic cancer, but even for those patients who undergo radical surgery and receive standard postoperative chemotherapy, the 5-year survival rate remains poor—only 30% [5,6,7]. Therefore, it is of certain clinical value to evaluate the postoperative prognosis of pancreatic cancer patients at an early stage.

Cholesterol is an important molecule in the human body, and has important physiological functions, such as composing cell membranes, transporting fat-soluble vitamins and synthesizing hormones [8]. Serum total cholesterol (TC) level is determined by both exogenous absorption level and endogenous production level, and is affected by various perioperative factors [9]. Meanwhile, studies have also reported that perioperative serum TC level has certain predictive value for postoperative prognosis in patients with gastric cancer, breast cancer, liver cancer and other malignant tumors. Higher perioperative serum TC level suggests better long-term prognosis and lower postoperative recurrence rate [10,11,12]. Its predictive value may be attributed to its close correlation with tumor biological behavior and perioperative nutritional status, perioperative immune function, oxidative stress level and secretion of inflammatory factors such as IL-6 [13]. As for pancreatic cancer, it has been reported that the occurrence and progression of pancreatic cancer are related to abnormal lipid absorption and metabolism [14]. Patients with pancreatic cancer usually suffer from malnutrition, major surgical trauma, severe postoperative systemic inflammation and perioperative complications that may affect the serum TC level. Therefore, serum TC level may reflect the postoperative prognosis of patients with pancreatic cancer and have potential predictive value [15,16]. Current studies mainly focus on the value of serum TC level in screening and early diagnosis of pancreatic cancer. No relevant studies have revealed its predictive value for postoperative prognosis in pancreatic cancer patients.

Our study aimed to evaluate the relationship between perioperative serum TC level and postoperative long-term prognosis of pancreatic cancer patients and explore its potential predictive value for postoperative long-term prognosis.

## 2. Methods

### 2.1. Patient Screening

**Figure 1:**
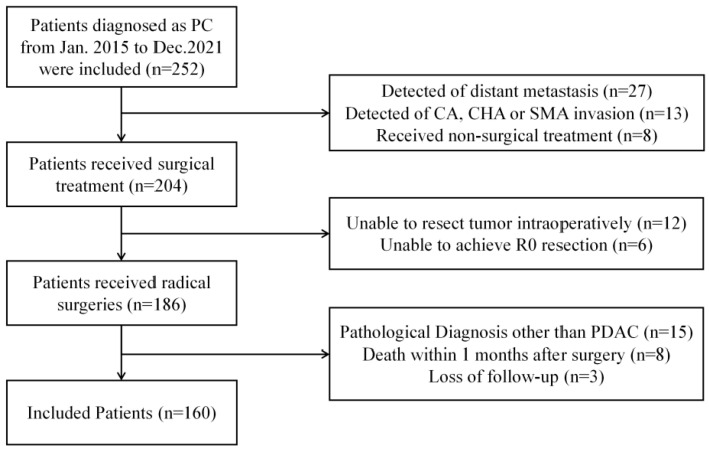
Flowchart of patient screening (PC: pancreatic cancer; CA: celiac axis; CHA: common hepatic artery; SMA: superior mesenteric artery; PDAC: pancreatic ductal adenocarcinoma).

We retrospectively analyzed patients with pancreatic cancer who received surgical treatment from January 2015 to December 2021 in the Hepatobiliary Surgery Department, Beijing Chaoyang Hospital. Patients that met the inclusion criteria were included in our research. A flowchart of patient selection is shown in Figure 1.

Inclusion criteria: (1) patients with pancreatic cancer who received surgical treatment from January 2015 to December 2021 in the Hepatobiliary Surgery Department, Beijing Chaoyang Hospital; (2) no restriction on age or gender; (3) no surgical contraindication, including abdominal arteries invasion and distant metastasis on preoperative evaluation; (4) achieved en bloc resection intraoperatively; (5) postoperative pathological confirmation of the diagnosis of pancreatic ductal adenocarcinoma and intraoperative R0 resection confirmed in postoperative pathology; (6) completeness of clinical and postoperative information.

Exclusion criteria: (1) unable to resect or achieve en bloc resection intraoperatively; (2) postoperative pathological examination other than pancreatic ductal adenocarcinoma; (3) postoperative pathological confirmation of R1 and R2 resection intraoperatively; (4) death within 2 months after surgery; (5) loss of postoperative follow-up data.

Informed consent of patients and their families were obtained for the surgical methods and treatment strategies of all patients in this research. The acquisition of clinical data of patients was approved by the Ethics Committee of Beijing Chaoyang Hospital (2020-D.-302).

### 2.2. Grouping

Serum TC level at five time points, which were preoperation, postoperative 3 days, postoperative 1 week, postoperative 4 weeks, and postoperative 8 weeks, were analyzed in our research. The preoperative serum TC level was obtained on the first day after admission. ROC curves of serum TC level at different time points and 1-year survival rate were drawn, from which the area under the curve (AUC) was calculated and the optimal cutoff value determined.

### 2.3. Data Analysis and Follow-Up

Preoperative general data, intraoperative data, and postoperative data of included patients were acquired from medical records and further compared. All patients were followed up at 1 and 3 months after surgery. Then, they were reexamined every 3 months in the first 2 years and thereafter every 6 months or until tumor recurrence or death. The result, of blood tests (blood routine, blood biochemistry, CA19-9) and imaging examination (abdominal enhanced CT, pulmonary CT), subsequent therapeutic schedule, postoperative tumor recurrence and survival were obtained and compared within these two groups.

### 2.4. Statistical Analysis

Measurement data are presented as means ± standard errors of the mean and compared with *t* tests if normally distributed. Measurement data with abnormal distribution are presented as medians (interquartile range) and were compared with rank-sum tests. Fisher analysis was applied in the comparison of categorical data if the theoretical frequency was less than 1 or sample size less than 40. Otherwise, chi-squared analysis was selected for the comparison. Survival outcomes were calculated using the Kaplan–Meier method and compared using the log-rank test. Variables that were significant on univariate analysis were included in a multivariate Cox proportional-hazard regression model. All statistical analyses were performed using SPSS (version 26.0; IBM Corporation, Armonk, NY, USA), with two-sided *p* values < 0.05 considered to be significant.

## 3. Results

### 3.1. Sample Characteristics

A total of 160 patients were included in our research: 89 male and 71 female (male:female = 1.3:1). The age of patients was 63.0 ± 10.1 years. The initial symptoms included abdominal pain (*n* = 61), jaundice (*n* = 73), and atypical digestive symptoms (*n* = 8), while those for the other 18 patients were found during physical examination. In sum, 53 patients (33.1%) had a history of diabetes. In 73 patients with jaundice, 36 received preoperative jaundice reduction treatment: 32 cases of percutaneous transhepatobiliary drainage (PTBD) and 4 cases of endoscopic retrograde cholangiopancreatography (ERCP). Characteristics of patients are shown in Table 1.

### 3.2. Perioperative Condition

The perioperative condition of the patients is shown in Table 2. A total of 12 patients received preoperative neoadjuvant chemotherapy. All patients underwent successful surgery and achieved en bloc resection intraoperatively. Seventy patients for which invasion of the portal vein system was suspected underwent venous resection and reconstruction intraoperatively. Blood loss was 500 (400–800) mL, and 62 patients (38.8%) received blood transfusion during surgeries. The operation time was 10 (8–12) h.

All patients were diagnosed with pancreatic ductal adenocarcinoma and achieved R0 resection based on postoperative pathological examination, including 101 cases of moderate differentiation (63.1%), 47 cases of poor differentiation (29.4%), and 12 cases of high differentiation (7.5%). Tumor size was 3.5 (2.5, 4.5) cm, and 100 patients (62.5%) had lymph node metastasis during postoperative pathological examination.

A total of 48 patients (32.0%) suffered from postoperative complications, including 9 cases (5.6%) of biochemical fistula, 7 cases (4.4%) of grade B pancreatic fistula, 4 cases (2.5%) of grade C pancreatic fistula, 15 cases (9.4%) of delayed gastric emptying, 12 cases (7.5%) of abdominal infection, 2 cases (1.3%) of abdominal hemorrhage, 3 cases (1.9%) of gastrointestinal bleeding, 2 cases (1.3%) of pulmonary infection, 1 case (0.6%) of biliary fistula, 1 case of portal venous embolism, and 1 case (0.6%) of pulmonary embolism. The median hospital stay of included patients after surgery was 19 (15–25) days.

### 3.3. Overall Prognosis

**Figure 2:**
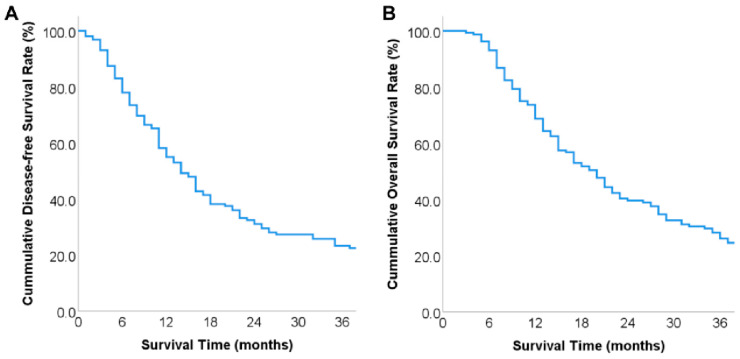
Long-term prognosis of patients: (**A**) overall disease-free survival curve of included patients; (**B**) overall survival curve of included patients.

Our follow-up ended in December 2022, during which 86 patients (53.8%) received 1 to 18 cycles of postoperative chemotherapy. Median disease-free survival (DFS) was 14 months and 1-, 2-, and 3-year DFS rates were 55.0%, 31.1%, and 22.5%, respectively (Figure 2A). Median overall survival (OS) was 20 months and 1-, 2-, and 3-year OS rates were 68.7%, 39.7%, and 26.1%, respectively (Figure 2B).

### 3.4. Grouping Situation

**Figure 3:**
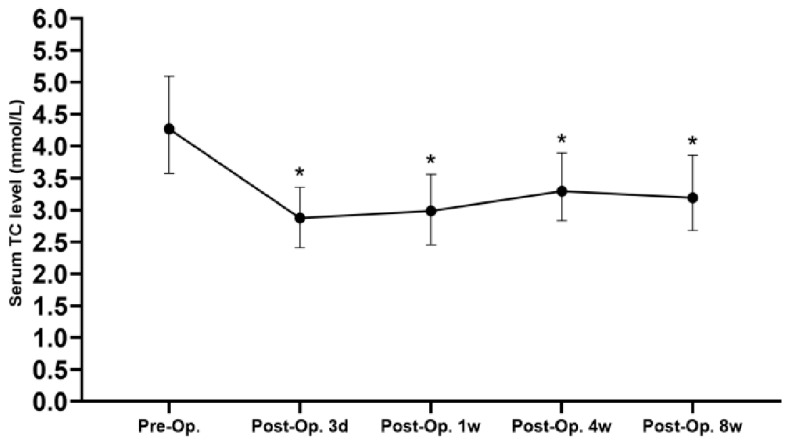
The variation tendency of serum TC perioperatively (*: compared with preoperative TC, *p* < 0.05, TC: total cholesterol, Preop.: preoperation, Postop.: postoperation).

**Figure 4:**
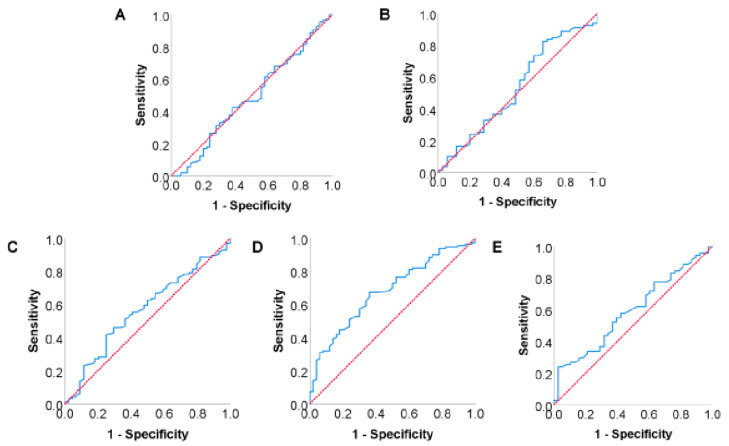
ROC curves between serum TC level at different time points and 1-year survival rate: (**A**) ROC curve between preoperative serum TC level and 1-year survival rate; (**B**) ROC curve between postoperative 3-day serum TC level and 1-year survival rate; (**C**) ROC curve between postoperative 1-week serum TC level and 1-year survival rate; (**D**) ROC curve between postoperative 4-week serum TC level and 1-year survival rate; (**E**) ROC curve between postoperative 8-week serum TC level and 1-year survival rate.

The variation tendency of the perioperative serum TC levels of included patients is shown in Figure 3, indicating that the serum TC level gradually recovered after a transient decline in the early stage after surgery. However, it was still significantly lower than the preoperative level, even after 8 weeks of recovery (*p* < 0.05). The ROC curves of serum TC level at different time points and 1 year survival were drawn and are shown in Figure 4. The AUC of the ROC curve between preoperative, postoperative 3 day, week 1, week 4, week 8 serum TC level, and 1-year survival rates were 0.487 (95% CI: 0.387–0.586), 0.534 (95% CI: 0.415–0.653), 0.566 (95% CI: 0.464–0.667), 0.679 (95% CI: 0.593–0.765) and 0.592 (95% CI: 0.483–0.701), respectively. Serum TC level at postoperative 4 weeks was selected as the subject of our research according to the AUC of ROC curves. The optimal cutoff value of postoperative 4-week serum TC level was calculated by ROC curve and determined as 3.095 mmol/L. Its sensitivity and specificity in predicting 1-year survival were 67.3% and 64.0%, respectively. Patients were then divided into low-TC (postoperative 4-week serum TC ≤ 3.095 mmol/L, *n* = 68) and high-TC groups (postoperative 4-week serum TC > 3.095 mmol/L, *n* = 92) based on the optimal cutoff value.

### 3.5. Comparison of Perioperative and Long-Term Prognostic Data of Total Patients Grouped by Postoperative Serum TC Level

**Figure 5:**
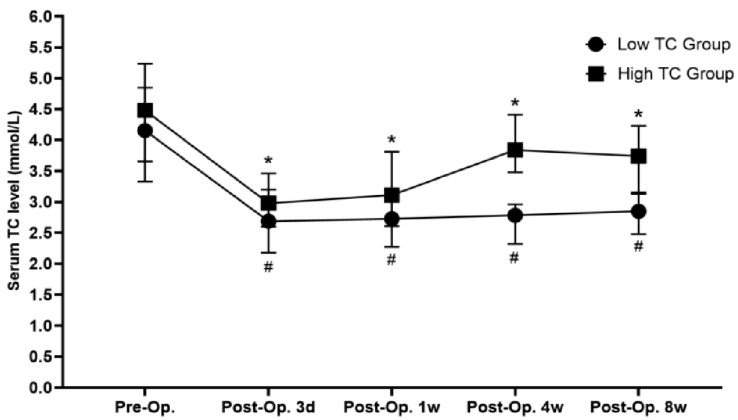
Variation in serum TC in the low-TC group and high-TC group perioperatively (*: compared with preoperative serum TC in the high-TC group, *p* < 0.05, #: compared with preoperative serum TC in the low-TC group, *p* < 0.05, TC: total cholesterol, Preop.: preoperation, Postop.: postoperation).

The comparison of perioperative general data between the low-TC group and high-TC group is shown in Table 3, indicating that the serum TC level at preoperation, postoperative 3 days, 1 week, and 8 weeks and postoperative 4-week serum albumin in the high-TC group were significantly higher than that of low-TC group (*p* < 0.05). The variation tendency of perioperative serum TC level in the low-TC group and high-TC group is shown in Figure 5. It could be observed that although the postoperative recovery of the serum TC level in the high-TC group was more rapid than that in the low-TC group, the postoperative serum TC level in both of these groups was significantly lower than that before surgery (*p* < 0.05). The comparison of major postoperative complications between the low-TC group and the high-TC group is shown in Table 4, indicating no statistical difference in postoperative mortality rate or incidence of postoperative complications between these groups (*p* > 0.05).

**Figure 6:**
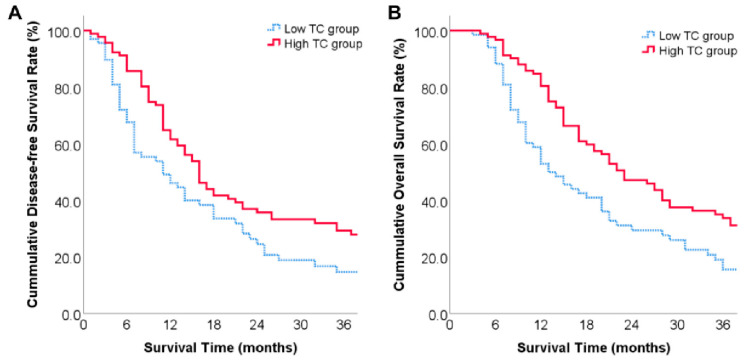
Long-term prognosis of low-TC group and high-TC group: (**A**) overall disease-free survival rate of low-TC group and high-TC group; (**B**) overall survival rate of low-TC group and high-TC group.

The median DFS of patients in the low-TC and high-TC groups was 11 months and 16 months, respectively, and DFS rates at 1, 2 and 3 years after surgery were 46.1%, 24.5%, 14.7% and 61.6%, and 35.8% and 29.3%, respectively (*p* = 0.041, Figure 6A). The median OS of patients in the low-TC group and high-TC group was 13 months and 23 months, respectively, and the OS rate at 1, 2, and 3 years after surgery was 52.9% and 29.4%, 15.6% and 80.4%, and 47.2% and 33.8%, respectively (*p* = 0.005, Figure 6B).

### 3.6. Analysis of Risk Factors for Postoperative Tumor Recurrence in Pancreatic Cancer Patients

In our research, postoperative tumor recurrence in pancreatic cancer patients was taken as a dependent variable and the perioperative data of patients including preoperative data (gender, age, diabetes, preoperative jaundice reduction treatment, preoperative total bilirubin, preoperative CA19-9, preoperative serum TC, preoperative serum albumin, preoperative serum choline esterase), intraoperative data (operative time, volume of blood loss, intraoperative blood transfusion), pathological data (pTNM stage, tumor location, peripancreatic invasion, degree of tumor differentiation, tumor diameter, lymph node metastasis, portal vein invasion) and postoperative data (postoperative complications, postoperative chemotherapy, serum TC at postoperative 3 days, 1 week, 4 weeks and 8 weeks, serum albumin at postoperative 4 weeks, serum choline esterase at postoperative 4 weeks) were taken as independent variables for univariate analysis. The results, as shown in Table 5, indicated that postoperative 4-week serum TC, degree of tumor differentiation, pTNM stage, tumor diameter and lymph node metastasis were potential risk factors for postoperative tumor recurrence and were further included in Cox proportional-hazard regression models for multivariate analysis. The results showed that degree of tumor differentiation (*RR* = 2.177, 95% CI: 1.478–3.209), pTNM stage (*RR* = 1.609, 95% CI: 1.023–2.529) and lymph node metastasis (*RR* = 1.637, 95% CI: 1.087–2.465) were independent risk factors for postoperative tumor recurrence in pancreatic cancer patients, as shown in Table 6. Patients with a higher degree of tumor differentiation, low pTNM stage and no lymph node metastasis tended to have a lower risk of postoperative tumor recurrence.

### 3.7. Analysis of Risk Factors for Postoperative Long-Term Survival in Pancreatic

#### Cancer Patients

In our research, postoperative long-term survival in pancreatic cancer patients was taken as a dependent variable and the perioperative data of patients, including preoperative data (gender, age, diabetes, preoperative jaundice reduction treatment, preoperative total bilirubin, preoperative CA19-9, preoperative serum TC, preoperative serum albumin, preoperative serum choline esterase), intraoperative data (operative time, volume of blood loss, intraoperative blood transfusion), pathological data (pTNM stage, tumor location, peripancreatic invasion, degree of tumor differentiation, tumor diameter, lymph node metastasis, portal vein invasion) and postoperative data (postoperative complications, postoperative chemotherapy, serum TC at postoperative 3 days, 1 week, 4 weeks and 8 weeks, serum albumin at postoperative 4 weeks, serum choline esterase at postoperative 4 weeks) were taken as independent variables for univariate analysis. The results shown in Table 7 indicated that serum TC at postoperative 4 weeks, degree of tumor differentiation, pTNM stage, tumor diameter and lymph node metastasis were potential risk factors for poor postoperative long-term prognosis and were further included in Cox proportional-hazard regression models for multivariate analysis. The results showed that serum TC at postoperative 4 weeks (*RR* = 0.663, 95% CI: 0.466–0.944), degree of tumor differentiation (*RR* = 2.054, 95% CI: 1.396–3.025), pTNM stage (*RR* = 1.595, 95% CI: 1.020–2.494) and lymph node metastasis (*RR* = 1.693, 95% CI: 1.127–2.544) were independent risk factors for poor postoperative long-term survival in patients with pancreatic cancer, as shown in Table 8. Patients with higher postoperative 4-week serum TC level, higher degree of tumor differentiation, low pTNM stage and no lymph node metastasis tended to have better postoperative long-term survival.

## 4. Discussion

Cholesterol is an important molecule in the human body, and plays an important role in composing cell membranes, maintaining cell functions, and acting as a precursor of hormones [8]. Serum cholesterol mainly comes from endogenous and exogenous pathways [17]. The exogenous pathway refers to the process in which cholesterol in food and bile acids secreted by the liver are absorbed in the small intestine by interacting with receptors such as NPC1L1 on the surface of the small intestinal villi, while the endogenous pathway refers to the synthesis of cholesterol by various nucleated cells using acetyl CoA produced by glucose metabolism and lipid metabolism as raw materials, of which about 50% is synthesized in the liver [18,19]. Therefore, serum TC is affected by many factors, which may be related to surgery, liver function, nutritional status and other factors [9]. Serum TC is also affected by tumor factors according to current research. It has been reported that serum TC is associated with the occurrence and prognosis of colon cancer, liver cancer, kidney cancer and other tumors [20,21,22]. Lipid metabolism reprogramming is common in pancreatic cancer tissue and has been identified as an important characteristic of pancreatic cancer. Cholesterol is essential for rapidly expanding pancreatic cancer cells due to its involvement in cell membrane synthesis, signal transduction and energy supply [14]. Current research has not only revealed the correlation between the progression and drug resistance of pancreatic cancer and cholesterol but also demonstrated that tumors increase the uptake of exogenous cholesterol by increasing LDL receptors on the surface of cancer cells so as to meet their high avidity for cholesterol [23]. Therefore, we hypothesized that perioperative serum TC level may be a potential indicator to predict the long-term postoperative prognosis of pancreatic cancer patients.

High serum TC level is considered one of the important protective factors for patients with malignant tumors [13]. Shin et al. retrospectively analyzed patients undergoing surgical treatment for gastric cancer and found that patients with higher preoperative serum TC level not only had higher postoperative DFS rate and OS rate but also had lower postoperative complication incidence [10]. Colhoun et al. found that the postoperative recurrence rate of hepatocellular carcinoma increased significantly when the preoperative serum TC level was less than 100 mg/dL in patients receiving liver transplantation for hepatocellular carcinoma [12]. The ability of cholesterol to reflect nutritional status and affect cellular immunity in pancreatic cancer patients may be the potential reason for its influence in postoperative prognosis. Currently, it has been reported that serum TC level is also one of the indicators of nutritional status and hypocholesterolemia can reflect the malnutrition status [24]. Serum TC level was also included in controlling nutritional status (COUNT) score to evaluate the nutritional status of patients [25]. In our research, serum albumin level, which is also acknowledged as an important index to reflect nutritional status, was significantly higher in the high-TC group compared with the low-TC group at 4 weeks after surgery, indicating a better nutritional status in the high-TC group. This result further confirmed the correlation between serum TC level and nutritional status of pancreatic cancer patients. Caused by many factors, including internal and external secretion insufficiency, occurrence of postoperative complications, and so on, malnutrition is commonly seen in pancreatic cancer patients and can lead to poor postoperative prognosis [26]. This may partly explain the prognostic effect of serum TC level. Current studies have demonstrated the prognostic value of preoperative COUNT score in pancreatic cancer patients and attributed its better predictive effect to the inclusion of preoperative serum TC level [27]. However, our results indicated no positive correlation between preoperative serum TC level and the prognosis of pancreatic cancer patients, which we consider to be related to biliary obstruction and cholestasis. For pancreatic cancer in the early stage, it performs relatively well, consumes less serum cholesterol, and causes mild malnutrition in patients. For those at an advance stage, it has poor biological behavior, in spite of severe malnutrition caused by tumor consumption. Serum TC level may remain at a high level due to cholesterol excretion disorders secondary to biliary obstruction and cholestasis caused by advanced tumors [28]. In addition, due to the important role of cholesterol in cell membrane synthesis and biological signal transmission, an appropriate level of cholesterol in cells is currently considered to play an important role in the maintenance of antitumor immune function. Sag et al. observed that high levels of intracellular cholesterol induced macrophages to polarize towards M1 macrophages with antitumor effects, rather than M2 macrophages with protumor effects [29]. Mundoon et al. found that the total number of serum lymphocytes, T cells and CD8^+^ T cells in the high-cholesterol group were significantly higher than those in the low-cholesterol group [30]. Yang et al. reported that inhibiting cholesterol esterification could increase the cholesterol content of cell membrane and improve the antitumor effect of CD8^+^ T cells [31]. As the exogenous source of intracellular cholesterol, serum TC level may affect antitumor immunity and may be one of the reasons for its influence on prognosis.

Researchers have considered serum TC level to be accumulative and that its changes can reflect disease progression and severity, which may explain the higher serum TC level in the high serum TC group than the low serum TC group at every time point perioperatively [9,32]. In this research, we observed a temporary decrease in serum TC level at the early stage after surgery, which may be related to the superimposed influence of tumor factors, preoperative nutritional status, surgical conditions and other factors. Upon comparison of general data between the low-TC and high-TC groups, we observed no statistical differences between intraoperative and postoperative conditions or pTNM stage and pathological condition of tumors, indicating that received radical resection not only eliminate the negative effects of tumors but also helped in recovery from surgery and postoperative complications after 4 weeks [26]. At that time, there are fewer confounding factors that can influence the serum TC level, which can render accurate postoperative conditions of pancreatic cancer patients. Taking the better AUC under the ROC curve into consideration, we decided to select the serum TC level at postoperative 4 weeks as the study object.

Surgical factors are important factors affecting serum TC levels. Akgün et al. observed a significant decrease in serum TC level after most surgeries [33]. Furthermore, Tabriz et al. retrospectively analyzed patients who underwent pylorus-preserving pancreaticoduodenectomy for pancreatic cancer and found that the serum TC level at 5 days after surgery was significantly lower than that at 2 days before surgery [34]. These findings are consistent with our result and may be attributed to intraoperative condition and postoperative inflammatory response. Sun et al. found that postoperative serum TC level was directly related to intraoperative blood loss, intravenous fluid volume, and hematocrit [35]. A negative correlation between serum TC levels and IL-6 levels in postoperative patients was reported by Akgün et al. [33]. However, postoperative serum TC level early after surgery is affected not only by surgical factors but also by other factors, including preoperative baseline serum TC level and postoperative fluid resuscitation, which may explain its inability to predict postoperative long-term prognosis of pancreatic cancer patients.

The occurrence of postoperative complications is also one of the factors influencing serum TC level. After retrospectively analyzing 70 patients with hilar cholangiocarcinoma, Chen et al. found that hypocholesterolemia on the first postoperative day was an independent risk factor for postoperative infectious complications [36]. Nuzzo et al. reported that postoperative serum TC level under 1.3–1.5 mmol/L often indicates the occurrence of postoperative sepsis after hepatopancreatobiliary surgery, and persistent hypocholesterolemia hints at more severe disease condition and worse prognosis [37]. Serum TC level decreased in the early postoperative period and increased after 7 days according to our findings. Complications after surgery, inflammation, synthesis disorders and other reasons will lead to a delay in recovery of serum TC level and finally cause a decline in serum cholesterol level at 4 weeks after surgery [38]. However, although the serum TC levels at 4 weeks after surgery was significantly lower in the low-TC group than that in the high-TC group, we found no statistical difference in the incidence of postoperative complications between these two groups. Differences in the severity of postoperative complications and the time of intervention may explain this result. Eight patients in the high-TC group and five in the low-TC group underwent reoperation due to various complications, with median time to surgical intervention 11 days and 15 days, respectively. Among infectious complications, six patients with abdominal infection in the low-TC group received further treatment—four with surgical debridement and two with puncture drainage, while in the high-TC group, only three of six patients with abdominal infection received surgical debridement and the remaining three had improved after conservative treatment. Early intervention and relatively mild complications in the high-TC group may be the reasons for the higher serum TC at 4 weeks after operation.

We also confirmed tumor differentiation degree, pTNM stage and lymph node metastasis as independent risk factors for postoperative tumor recurrence and long-term prognosis, consistent with previous research [39,40]. Our results further confirmed the prognostic value of these indicators in pancreatic cancer patients.

Our research preliminarily confirmed the prognostic value of postoperative 4-week serum TC level in predicting the postoperative prognosis of pancreatic cancer patients and provided a potential marker to evaluate the prognosis of pancreatic cancer at an early stage after surgery. Since serum TC level is an easily accessible index, our study is practical in the clinic. In the future, a comprehensive prediction model of serum lipid profile on the basis of this research can be established to better predict the postoperative prognosis of pancreatic cancer patients. Postoperative 4-week serum TC level may also have certain value in guiding comprehensive antitumor treatment so as to improve the prognosis of pancreatic cancer patients, which requires further prospective research to verify. Therefore, our research is of certain clinical value and may benefit the prognosis of pancreatic cancer patients in the future.

Our research has some limitations. First, this was a retrospective study and the reported predictive value of serum TC in evaluating long-term postoperative prognosis requires further prospective studies to validate our conclusion. Second, further research is necessary to determine more accurate cutoff values to better predict long-term outcomes in postoperative prognosis of patients with pancreatic cancer. Third, postoperative management measures to improve the prognosis of pancreatic cancer patients were not identified. Whether patients with low postoperative 4-week serum TC can benefit from sufficient postoperative nutritional support and aggressive chemotherapy requires further research.

## 5. Conclusions

In conclusion, our research showed that serum TC level significantly decreases at an early stage after surgery and gradually recovers. We also conclude that although higher serum TC at postoperative 4 weeks is not an independent risk factor for postoperative tumor recurrence, it is still a protective factor for a better long-term prognosis in pancreatic cancer patients, which may be an effective predictor of postoperative prognosis of patients with pancreatic cancer.

## Figures and Tables

**Table 1:** Characteristics of the 160 pancreatic cancer patients.

Characteristics	Overall (*n* = 160)
Gender, *n* (%)	
Male	89 (55.6%)
Female	71 (44.4%)
Age (years)	63.0 ± 10.1
Initial symptoms, *n* (%)	
Jaundice	73 (45.6%)
Abdominal pain	61 (38.1%)
Atypical digestive symptoms	8 (5.0%)
No relevant symptoms	18 (11.3%)
Diabetes, *n* (%)	53 (33.1%)
Preoperative jaundice reduction treatment, *n* (%)	
PTBD	32 (20.0%)
ERCP	4 (2.5%)

PTBD: percutaneous transhepatobiliary drainage; ERCP: endoscopic retrograde cholangiopancreatography.

**Table 2:** Perioperative condition of the 160 pancreatic cancer patients.

Characteristics	Overall (*n* = 160)
Neoadjuvant chemotherapy, *n* (%)	12 (7.5%)
Portal vein system invasion, *n* (%)	70 (43.8%)
Intraoperative blood loss (mL)	500 (400–800)
Operation time (h)	10 (8–12)
Intraoperative blood transfusion, *n* (%)	62 (38.8%)
Tumor differentiation, *n* (%)	
Poor differentiation	47 (29.4%)
Moderate differentiation	101 (63.1%)
High differentiation	12 (7.5%)
Tumor size (cm)	3.5 (2.5, 4.5)
Lymph node metastasis, *n* (%)	100 (62.5%)
Postoperative complications, *n* (%)	48 (30.0%)
Delayed gastric emptying, *n* (%)	15 (9.4%)
Abdominal infection, *n* (%)	12 (7.5%)
Pancreatic fistula, *n* (%)	
Biochemical fistula	9 (5.6%)
Grade B fistula	7 (4.4%)
Grade C fistula	4 (2.5%)
Gastrointestinal bleeding, *n* (%)	3 (1.9%)
Abdominal hemorrhage, *n* (%)	2 (1.3%)
Pulmonary infection, *n* (%)	2 (1.3%)
Biliary fistula, *n* (%)	1 (0.6%)
Portal venous embolism, *n* (%)	1 (0.6%)
Pulmonary embolism, *n* (%)	1 (0.6%)

**Table 3:** Comparison of perioperative general data between low-TC group and high-TC group.

Variables	Low-TC Group (*n* = 68)	High-TC Group (*n* = 92)	*p* Value
Gender (Male/Female)	39/29	50/42	0.705
Age (years)	62.3 ± 10.4	63.7 ± 10.0	0.399
Diabetes (Yes/No)	25/43	28/64	0.400
Preoperative jaundice reduction treatment (Yes/No)	16/52	20/72	0.789
Preoperative serum TB (μmol/L)	47.2 (13.6, 144.3)	30.3 (10.2, 127.8)	0.187
Preoperative serum GGT (U/L)	224.0 (25.0, 496.3)	134.0 (23.5, 547.0)	0.466
Preoperative serum CA19-9 (U/L)	252.8 (56.0, 1127.0)	175.6 (33.0, 634.0)	0.114
Preoperative serum ChE (U/L)	7785.0 (5487.5, 12,040.0)	8393.5 (4859.0, 11,250.3)	0.691
Preoperative serum albumin (g/L)	37.0 ± 4.6	36.4 ± 5.4	0.439
Postoperative 4-week serum ChE (U/L)	8056.5 (5254.8, 10,656.5)	9358.0 (5656.3, 11,006.5)	0.385
Postoperative 4-week serum albumin (g/L)	34.9 ± 4.7	36.7 ± 5.4	0.029
Preoperative serum TC (mmol/L)	4.16 (3.33, 4.85)	4.51 (3.66, 5.23)	0.033
Postoperative 3 days serum TC (mmol/L)	2.69 (2.18, 3.20)	2.98 (2.60, 3.46)	0.024
Postoperative 1 week serum TC (mmol/L)	2.73 (2.28, 3.20)	3.11 (2.61, 3.81)	0.003
Postoperative 8-week serum TC (mmol/L)	2.85 (2.48, 3.13)	3.74 (3.16, 4.23)	0.000
Intraoperative blood loss (mL)	500 (400, 800)	500 (400, 775)	0.242
Intraoperative blood transfusion (Yes/No)	31/37	31/61	0.127
Operation time (hours)	10.3 ± 3.2	9.6 ± 2.7	0.105
pTNM stage (I&II/III)	49/19	74/18	0.214
Tumor location (pancreatic head/pancreatic neck and tail)	55/13	64/28	0.105
Peri-pancreatic invasion (Yes/No)	65/3	89/3	0.705
Tumor diameter (cm)	3.5 (2.5, 4.7)	3.5 (2.4, 4.2)	0.684
Tumor differentiation (Poor/moderate & high)	20/48	27/65	0.993
Portal vein system invasion (Yes/No)	34/34	36/56	0.171
Neoadjuvant chemotherapy (Yes/No)	5/63	7/85	0.952
Lymph nodes metastasis (Yes/No)	46/22	54/38	0.248
Postoperative chemotherapy (Yes/No)	36/32	50/42	0.860

TC: total cholesterol; TB: total bilirubin; CA19-9: carbohydrate antigen 19-9; GGT: gamma-glutamyl transpeptidase; ChE: choline esterase.

**Table 4:** Comparison of major postoperative complications between low-TC group and high-TC group.

Variables	Low-TC Group (*n* = 68)	High-TC Group (*n* = 92)	*p* Value
Postoperative hospital stay (days)	19 (15, 28)	19 (13, 25)	0.265
Postoperative complications	19	29	0.722
Biochemical fistula	2	7	0.358
Pancreatic fistula			
Grade B	3	4	1.000
Grade C	2	2	1.000
Delayed gastric emptying	6	9	0.837
Abdominal infection	6	6	0.585
Pulmonary infection	2	1	0.748
Abdominal hemorrhage	1	1	1.000
Gastrointestinal bleeding	0	3	0.614

TC: total cholesterol.

**Table 5:** Univariate analysis of postoperative tumor recurrence in pancreatic cancer patients.

Variables	Number (*n* = 160)	1-Year DFS Rate (%)	3-Year DFS Rate (%)	*χ*^2^ Value	*p* Value
Gender				0.660	0.416
Male	89	47.3	20.4		
Female	71	64.5	25.2		
Age (years)				0.015	0.904
≤60	60	59.5	18.5		
>60	100	52.3	25.1		
Diabetes				0.045	0.833
Yes	53	49.4	22.2		
No	107	57.7	22.8		
Preoperative jaundice reduction treatment				0.000	0.994
Yes	36	47.2	18.3		
No	124	57.3	22.7		
Preoperative serum TB (μmol/L)				0.094	0.760
≤21	70	57.4	23.9		
>21	90	53.3	20.2		
Preoperative serum CA19-9 (U/mL)				0.044	0.834
≤37	40	52.5	24.2		
>37	120	55.9	20.9		
Preoperative serum albumin (g/L)				3.574	0.059
≤40	119	51.5	18.7		
>40	41	65.2	30.7		
Postoperative 4-week serum albumin (g/L)				3.367	0.067
≤40	124	50.7	19.7		
>40	36	69.4	32.2		
Preoperative serum ChE (U/L)				0.106	0.745
≤4900	38	57.9	23.0		
>4900	122	52.4	21.3		
Postoperative 4-week serum ChE (U/L)				0.002	0.965
≤4900	33	45.5	22.5		
>4900	127	57.6	22.4		
Preoperative serum TC (mmol/L)				0.189	0.663
≤4.535	91	56.4	24.7		
>4.535	69	53.1	19.6		
Postoperative 3 days serum TC (mmol/L)				1.140	0.286
≤2.375	60	46.2	20.6		
>2.375	100	59.3	23.8		
Postoperative 1 week serum TC (mmol/L)				0.039	0.843
≤3.145	89	51.3	23.2		
>3.145	71	59.7	21.7		
Postoperative 4-week serum TC (mmol/L)				4.172	0.041
≤3.095	68	46.1	14.7		
>3.095	92	61.6	27.9		
Postoperative 8-week serum TC (mmol/L)				0.339	0.560
≤4.185	119	53.2	22.8		
>4.185	41	60.3	21.4		
Operation time (hours)				1.373	0.241
≤8	58	57.5	33.7		
>8	102	53.5	18.1		
Intraoperative blood loss (mL)				1.058	0.304
≤800	90	58.9	25.2		
>800	70	49.9	19.0		
Intraoperative blood transfusion				1.829	0.176
Yes	62	47.4	16.0		
No	98	59.8	27.5		
Tumor differentiation				18.268	0.000
Poor	47	29.8	7.2		
Moderate and high	113	65.7	29.1		
pTNM stage				16.692	0.000
I&II	123	61.1	29.6		
III	37	35.1	0.0		
Peripancreatic invasion				0.951	0.329
Yes	6	33.3	16.7		
No	154	55.9	22.0		
Tumor location				0.015	0.902
Pancreatic head	119	55.5	21.4		
Pancreatic neck and tail	41	53.6	23.0		
Tumor diameter (cm)				7.440	0.006
≤4	112	58.5	27.5		
>4	48	44.3	6.6		
Lymph node metastasis				12.105	0.001
Yes	100	44.6	14.8		
No	60	72.7	35.5		
Portal vein system invasion				1.889	0.169
Yes	70	51.4	17.3		
No	90	57.8	26.9		
Postoperative complications				0.793	0.373
Yes	48	44.8	21.5		
No	112	59.4	22.9		
Postoperative chemotherapy				0.005	0.945
Yes	86	59.8	20.0		
No	74	49.4	23.2		

DFS: disease-free survival; TB: total bilirubin; CA19-9: carbohydrate antigen 19-9; TC: total cholesterol; ChE: choline esterase.

**Table 6:** Multivariate analysis of postoperative tumor recurrence in pancreatic cancer patients.

Variables	*RR* Value	95% CI	*p* Value
Postoperative 4-week serum TC	0.782	0.549–1.115	0.175
Tumor differentiation	2.177	1.478–3.209	0.000
pTNM stage	1.609	1.023–2.529	0.040
Tumor diameter	1.189	0.775–1.825	0.429
Lymph node metastasis	1.637	1.087–2.465	0.018

TC: total cholesterol; CI: confidence interval.

**Table 7:** Univariate analysis of postoperative long-term survival in pancreatic cancer patients.

Variables	Number (*n* = 160)	1-Year OS Rate (%)	3-Year OS Rate (%)	*χ*^2^ Value	*p* Value
Gender				0.335	0.563
Male	89	66.3	25.4		
Female	71	71.8	26.9		
Age (years)				0.070	0.792
≤60	60	71.7	19.5		
>60	100	67.0	30.5		
Diabetes				0.361	0.548
Yes	53	67.9	28.8		
No	107	69.2	24.9		
Preoperative jaundice reduction treatment				0.497	0.481
Yes	36	55.6	20.7		
No	124	72.6	26.6		
Preoperative serum TB (μmol/L)				0.020	0.887
≤21	70	72.9	31.4		
>21	90	65.6	26.0		
Preoperative serum CA19-9 (U/mL)				0.268	0.605
≤37	40	70.0	32.1		
>37	120	68.3	22.4		
Preoperative serum albumin (g/L)				2.255	0.133
≤40	119	67.2	23.5		
>40	41	73.2	34.5		
Postoperative 4-week serum albumin (g/L)				2.031	0.154
≤40	124	66.1	23.8		
>40	36	77.8	25.5		
Preoperative serum ChE (U/L)				0.045	0.832
≤4900	38	65.8	24.7		
>4900	122	67.2	26.5		
Postoperative 4-week serum ChE (U/L)				0.068	0.794
≤4900	33	60.6	22.8		
>4900	127	70.9	26.9		
Preoperative serum TC (mmol/L)				0.000	0.990
≤4.535	91	67.0	28.3		
>4.535	69	71.0	23.5		
Postoperative 3 days serum TC (mmol/L)				1.467	0.226
≤2.375	60	58.3	19.9		
>2.375	100	75.0	28.2		
Postoperative 1 week serum TC (mmol/L)				0.421	0.517
≤3.145	89	62.9	26.0		
>3.145	71	76.1	24.6		
Postoperative 4-week serum TC (mmol/L)				7.817	0.005
≤3.095	68	52.9	15.6		
>3.095	92	80.4	33.8		
Postoperative 8-week serum TC (mmol/L)				2.304	0.129
≤4.185	119	63.9	25.6		
>4.185	41	82.9	43.1		
Operation time (hours)				1.932	0.165
≤8	58	75.9	36.6		
>8	102	64.9	20.8		
Intraoperative blood loss (mL)				0.912	0.340
≤500	90	74.4	29.6		
>500	70	61.4	21.6		
Intraoperative blood transfusion				1.997	0.158
Yes	62	62.9	17.9		
No	98	72.4	30.9		
Tumor differentiation				14.154	0.000
Poor	47	48.9	11.4		
Moderate & High	113	77.0	32.4		
pTNM stage				16.079	0.000
I&II	123	71.5	31.3		
III	37	59.5	5.9		
Extrapancreatic invasion				0.751	0.386
Yes	6	50.0	16.7		
No	154	68.8	26.5		
Tumor location				0.255	0.613
Pancreatic head	119	65.5	24.9		
Pancreatic neck and tail	41	70.7	30.2		
Tumor diameter (cm)				7.028	0.008
≤4	112	72.0	32.6		
>4	48	59.5	8.1		
Lymph node metastasis				14.425	0.000
Yes	100	62.0	17.6		
No	60	80.0	40.3		
Portal vein system invasion				3.255	0.071
Yes	70	61.4	20.7		
No	90	74.4	30.4		
Postoperative complications				0.230	0.631
Yes	48	64.6	27.1		
No	112	70.5	25.5		
Postoperative chemotherapy				0.173	0.677
Yes	86	75.6	22.4		
No	74	60.8	31.3		

(OS: overall survival; TB: total bilirubin; CA19-9: carbohydrate antigen 19-9; TC: total cholesterol; ChE: choline esterase).

**Table 8:** Multivariate analysis of postoperative long-term survival in pancreatic cancer patients.

Variables	*RR* Value	95% CI	*p* Value
Postoperative 4-week serum TC	0.663	0.466–0.944	0.023
Tumor differentiation	2.054	1.396–3.025	0.000
pTNM stage	1.595	1.020–2.494	0.041
Tumor diameter	1.259	0.844–1.878	0.260
Lymph node metastasis	1.693	1.127–2.544	0.011

TC: total cholesterol; CI: confidence interval.

## Data Availability

The datasets used and/or analyzed during the current study available from the corresponding authors on reasonable request.
